# Derivation and validation of a novel risk assessment tool to identify children aged 2–59 months at risk of hospitalised pneumonia-related mortality in 20 countries

**DOI:** 10.1136/bmjgh-2021-008143

**Published:** 2022-04-15

**Authors:** Chris A Rees, Tim Colbourn, Shubhada Hooli, Carina King, Norman Lufesi, Eric D McCollum, Charles Mwansambo, Clare Cutland, Shabir Ahmed Madhi, Marta Nunes, Joseph L Mathew, Emmanuel Addo-Yobo, Noel Chisaka, Mumtaz Hassan, Patricia L Hibberd, Prakash M Jeena, Juan M Lozano, William B MacLeod, Archana Patel, Donald M Thea, Ngoc Tuong Vy Nguyen, Cissy B Kartasasmita, Marilla Lucero, Shally Awasthi, Ashish Bavdekar, Monidarin Chou, Pagbajabyn Nymadawa, Jean-William Pape, Glaucia Paranhos-Baccala, Valentina S Picot, Mala Rakoto-Andrianarivelo, Vanessa Rouzier, Graciela Russomando, Mariam Sylla, Philippe Vanhems, Jianwei Wang, Rai Asghar, Salem Banajeh, Imran Iqbal, Irene Maulen-Radovan, Greta Mino-Leon, Samir K Saha, Mathuram Santosham, Sunit Singhi, Sudha Basnet, Tor A Strand, Shinjini Bhatnagar, Nitya Wadhwa, Rakesh Lodha, Satinder Aneja, Alexey W Clara, Harry Campbell, Harish Nair, Jennifer Falconer, Shamim A Qazi, Yasir B Nisar, Mark I Neuman

**Affiliations:** 1 Division of Pediatric Emergency Medicine, Emory University School of Medicine, Children’s Healthcare of Atlanta, Atlanta, Georgia, USA; 2 Institute for Global Health, University College London, London, UK; 3 Section of Pediatric Emergency Medicine, Texas Children’s Hospital, Baylor College of Medicine, Houston, Texas, USA; 4 Department of Global Public Health, Karolinska Institutet, Stockholm, Sweden; 5 Acute Respiratory Illness Unit, Government of Malawi Ministry of Health, Lilongwe, Malawi; 6 Global Program in Respiratory Sciences, Eudowood Division of Pediatric Respiratory Sciences, Department of Pediatrics, Johns Hopkins School of Medicine, Baltimore, Maryland, USA; 7 South African Medical Research Council: Vaccines and Infectious Diseases Analytics Research Unit, School of Pathology, Faculty of Health Sciences, University of the Witwatersrand, Johannesburg-Braamfontein, South Africa; 8 Advanced Pediatrics Centre, Postgraduate Institute of Medical Education and Research, Chandigarh, India; 9 Department of Pediatrics, Komfo Anokye Teaching Hospital, Kumasi, Ghana; 10 World Bank, World Bank, Washington, District of Columbia, USA; 11 Department of Pediatrics, Children’s Hospital, Islamabad, Pakistan; 12 Department of Global Health, Boston University School of Public Health, Boston, Massachusetts, USA; 13 Department of Paediatrics and Child Health, University of KwaZulu-Natal Nelson R Mandela School of Medicine, Durban, South Africa; 14 Division of Medical and Population Health Science Education and Research, Florida International University, Miami, Florida, USA; 15 Lata Medical Research Foundation, Nagpur and Datta Meghe Institute of Medical Sciences, Sawangi, India; 16 Department of Pediatrics, Children Hospital No 1, Ho Chi Minh City, Viet Nam; 17 Department of Child Health, Faculty of Medicine, Universitas Padjadjaran, Bandung, Indonesia; 18 Department of Pediatrics, Research Institute for Tropical Medicine, Muntinlupa City, Philippines; 19 Department of Pediatrics, King George's Medical University, Lucknow, Uttar Pradesh, India; 20 Department of Pediatrics, KEM Hospital Pune, Pune, India; 21 Rodolph Mérieux Laboratory, Faculty of Medicine, University of Health Sciences, Phnom Penh, Cambodia; 22 Department of Pediatrics, Mongolian Academy of Sciences, Ulaanbaatar, Mongolia; 23 Department of Pediatrics, GHESKIO, Port-au-Prince, Haiti; 24 Pediatrics, Fondation Merieux, Lyon, France; 25 Centre d'Infectiologie, Charles Mérieux, Antanarivo, Madagascar; 26 Departamento de Biología Molecular y Genética, Instituto de Investigaciones en Ciencias de la Salud, Asuncion, Paraguay; 27 Department of Pediatrics, Gabriel Touré University Hospital Center, Bamako, Mali; 28 Unité d'Hygiène, Epidémiologie, Infectiovigilance et Prévention, Hospices Civils de Lyon, Lyon, France; 29 MOH Key Laboratory of Systems Biology of Pathogens and Dr Christophe Mérieux Laboratory, Chinese Academy of Medical Sciences & Peking Union, Beijing, China; 30 Department of Paediatrics, Rawalpindi Medical College, Rawalpindi, Pakistan; 31 Department of Pediatrics, Sana’a University, Sana’a, Yemen; 32 Department of Pediatrics, Nishtar Medical College, Multan, Pakistan; 33 Division de Investigacion Insurgentes, Instituto Nactional de Pediatria, Mexico City, Mexico; 34 Infectious Diseases, Children's Hospital Dr Francisco de Ycaza Bustamante, Guayaquil, Ecuador; 35 Child Health Research Foundation, Dhaka Shishu Hosp, Dhaka, Bangladesh; 36 International Vaccine Access Center (IVAC), Department of International Health, Johns Hopkins University Bloomberg School of Public Health, Baltimore, Maryland, USA; 37 Department of Pediatrics, Medanta, The Medicity, Gurgaon, India; 38 Department of Pediatrics, Tribhuvan University Institute of Medicine, Kathmandu, Nepal; 39 Department of Research, Innlandet Hospital Trust, Lillehammer, Norway; 40 Department of Maternal and Child Health, Translational Health Science and Technology Institute, Faridabad, India; 41 Department of Pediatrics, All India Institute of Medical Sciences, New Delhi, India; 42 Department of Pediatrics, Sharda University School of Medical Sciences and Research, Greater Noida, Uttar Pradesh, India; 43 Central American Region, Centers for Disease Control and Prevention, Atlanta, Georgia, USA; 44 Population Health Sciences and Informati, The University of Edinburgh, Edinburgh, UK; 45 Centre for Global Health, Usher Institute, The University of Edinburgh, Edinburgh, Scotland; 46 Department of Maternal, Newborn, Child, and Adolescent Health (Retired), World Health Organization, Geneva, Switzerland; 47 Department of Maternal, Newborn, Child and Adolescent Health and Ageing, World Health Organization, Geneva, Switzerland; 48 Division of Emergency Medicine, Boston Children's Hospital, Harvard Medical School, Boston, Massachusetts, USA

**Keywords:** Pneumonia, Paediatrics

## Abstract

**Introduction:**

Existing risk assessment tools to identify children at risk of hospitalised pneumonia-related mortality have shown suboptimal discriminatory value during external validation. Our objective was to derive and validate a novel risk assessment tool to identify children aged 2–59 months at risk of hospitalised pneumonia-related mortality across various settings.

**Methods:**

We used primary, baseline, patient-level data from 11 studies, including children evaluated for pneumonia in 20 low-income and middle-income countries. Patients with complete data were included in a logistic regression model to assess the association of candidate variables with the outcome hospitalised pneumonia-related mortality. Adjusted log coefficients were calculated for each candidate variable and assigned weighted points to derive the Pneumonia Research Partnership to Assess WHO Recommendations (PREPARE) risk assessment tool. We used bootstrapped selection with 200 repetitions to internally validate the PREPARE risk assessment tool.

**Results:**

A total of 27 388 children were included in the analysis (mean age 14.0 months, pneumonia-related case fatality ratio 3.1%). The PREPARE risk assessment tool included patient age, sex, weight-for-age z-score, body temperature, respiratory rate, unconsciousness or decreased level of consciousness, convulsions, cyanosis and hypoxaemia at baseline. The PREPARE risk assessment tool had good discriminatory value when internally validated (area under the curve 0.83, 95% CI 0.81 to 0.84).

**Conclusions:**

The PREPARE risk assessment tool had good discriminatory ability for identifying children at risk of hospitalised pneumonia-related mortality in a large, geographically diverse dataset. After external validation, this tool may be implemented in various settings to identify children at risk of hospitalised pneumonia-related mortality.

**What is already known on this topic**

- In external validation of existing risk assessment tools to identify children at risk of hospitalised pneumonia-related mortality in varied settings, only the Respiratory Index of Severity in Children–Malawi score demonstrated fair discriminatory value (area under the curve (AUC) 0.75, 95% CI 0.74 to 0.77), while the Respiratory Index of Severity in Children score and a modified Pneumonia Etiology Research for Child Health score had limited discriminatory value (AUC 0.66, 95% CI 0.58 to 0.73, and AUC 0.55, 95% CI 0.37 to 0.73, respectively).

**What this study adds**

- Using data from 27 388 children in 20 low-income and middle-income countries, the Pneumonia Research Partnership to Assess WHO Recommendations (PREPARE) risk assessment tool was developed to identify children aged 2–59 months at risk of hospitalised pneumonia-related mortality across various settings.
- The PREPARE risk assessment tool had good discriminatory value when internally validated (AUC 0.83, 95% CI 0.81 to 0.84) and incorporates practical and commonly recorded clinical parameters to identify children at risk of hospitalised pneumonia-related mortality in various settings (ie, patient age, sex, weight-for-age z-score, body temperature, respiratory rate, unconsciousness or decreased level of consciousness, convulsions, cyanosis and hypoxaemia at baseline).

**How this study might affect research, practice and/or policy**

- After external validation, the PREPARE risk assessment tool may be implemented in various hospital settings in low-income and middle-income countries to identify children at risk of hospitalised pneumonia-related mortality.
- The impact of the implementation of existing risk assessment tools to identify children at risk of hospitalised pneumonia-related mortality must be compared with routine clinical care.

## Introduction

 Pneumonia is the leading cause of mortality among children 1–59 months of age, causing more than 800 000 deaths in this age group every year worldwide.^1^,^3^ Four risk assessment tools have been developed to identify children at risk of hospitalised pneumonia-related mortality in sub-Saharan Africa, South Asia and Southeast Asia.^4^,^7^ These risk assessment tools have important limitations, including the use of variables that are not routinely collected in clinical practice,^5^ being limited to single sites^4^,^6^ and the reliance on auscultatory findings with variable inter-rater reliability.^4 6^ Additionally, these risk assessment tools have not been widely implemented; thus, their potential to reduce hospitalised pneumonia-related mortality among children is unclear.^8^

We recently validated three of these risk assessment tools in a large, globally representative dataset using the demographic and clinical features of children at the time of admission.^9^ In that external validation, only the Respiratory Index of Severity in Children–Malawi (RISC-Malawi) score demonstrated fair discriminatory value (area under the curve (AUC) 0.75, 95% CI 0.74 to 0.77), while the Respiratory Index of Severity in Children (RISC) score and a modified Pneumonia Etiology Research for Child Health (PERCH) score had limited discriminatory value in identifying hospitalised children at risk of pneumonia-related mortality (AUC 0.66, 95% CI 0.58 to 0.73%, and AUC 0.55, 95% CI 0.37 to 0.73, respectively). The suboptimal performance of these prediction rules when applied externally raises questions on whether a novel tool incorporating different combinations of widely available clinical indicators could perform better across broad settings. Such a risk assessment tool should incorporate practical and commonly recorded clinical parameters to facilitate broad use and improved recognition of children at risk of hospitalised pneumonia-related mortality.

Given the limited discriminatory ability of prior risk assessment tools for pneumonia-related mortality when applied externally, our objective was to derive and validate a novel, widely applicable, risk assessment tool to identify children aged 2–59 months at risk of hospitalised pneumonia-related mortality. A risk assessment tool that is not region-specific may be useful to guide clinical care for children at greatest risk of hospitalised pneumonia-related mortality across settings.

## Methods

### Study design

We used the World Health Organization’s (WHO) Pneumonia Research Partnership to Assess WHO Recommendations (PREPARE) dataset to derive and validate a novel risk assessment tool for hospitalised pneumonia-related mortality among children 2–59 months of age across many global settings. We have described the details of the construction of the PREPARE dataset previously.^10^ Briefly, the PREPARE dataset includes primary data of individual patients from 30 study groups comprising 41 datasets of children evaluated for pneumonia in studies conducted from 1994 to 2014 in over 20 low-income and middle-income countries in Asia, Africa and Latin America, as well as the USA and Australia. We adhered to the Transparent Reporting of a Multivariable Prediction Model for Individual Prognosis or Diagnosis guidelines.^11^

### Patient and public involvement statement

The development of the research question was informed by the large burden of pneumonia-related mortality among children worldwide. Patients neither were advisers in this study nor were involved in the design, recruitment or conduct of the study. Results of this study will be made publicly available through open-access publication where study participants may access them.

### Study population

As pneumonia is the leading cause of mortality among children aged 1–59 months and pneumonia is defined differently in infants aged <2 months,^12 13^ we restricted the derivation and validation of our risk assessment tool to infants and children aged 2–59 months. We also restricted our analysis to studies that included hospitalised patients as our outcome was in-hospital mortality in children with suspected pneumonia (ie, pneumonia-related mortality). We excluded community-based studies, hospital-based studies that did not report survival data and any deaths that occurred outside the hospital. Pneumonia was defined according to the *2013 WHO Pocket Book of Hospital Care for Children* (ie, based on age-adjusted tachypnoea, presence of lower chest indrawing, general danger signs or signs of respiratory distress including head nodding/bobbing, nasal flaring or grunting in children with a cough or difficulty breathing).^14^

### Candidate variables

All candidate variables were selected *a priori*. As hypoxaemia has been shown to be highly predictive of pneumonia-related mortality among children^15^,^17^ and to avoid potential selection bias, we prioritised the inclusion of studies within the PREPARE dataset that had oxygen saturation (SpO_2_) measurements in at least 70% of he participants. Other candidate variables including age, sex, weight-for-age z-score, temperature, respiratory rate, presence of chest indrawing, unconsciousness/decreased consciousness, convulsions and cyanosis were selected based on the results of a systematic review evaluating risk factors for pneumonia-related mortality in children <5 years of age,^18^ prior clinical prediction models,^4^,^7^ as well as availability of data in the PREPARE dataset. Furthermore, hypoxaemia, presence of chest indrawing, unconsciousness or decreased consciousness, convulsions and cyanosis are signs and symptoms of severe pneumonia or danger signs according to the *2013 WHO Pocket Book of Hospital Care for Children*.^14^ Studies in the PREPARE dataset with >25% missing data points were excluded from our analysis to reduce selection bias. We did not include apnoea, gasping, grunting, nasal flaring, head nodding or stridor, which are general danger signs in the *2013 WHO Pocket Book of Hospital Care for Children*^14^ due to high levels of missing data.

We defined tachypnoea as 0–9, 10–19 and >20 breaths/min above age-specific cutoffs (ie, >50 breaths/min for children aged 2–11 months and >40 breaths/min for children aged 12–59 months).^12 14^ We categorised weight-for-age z-scores as <−3 for severe malnutrition, −3 to −2 for moderate malnutrition and >−2 for normal weight^19^; temperature as <35.5°C for hypothermia, 35.5°C to 37.9°C for normothermia and ≥38°C for fever^12^; and SpO_2_ as <90% for severe hypoxaemia, 90%–92% for mild hypoxaemia, and 93%–100% as normal.^20^ These continuous variables were converted into categorical variables based on recommended thresholds before developing our model to facilitate the use of this risk assessment tool in clinical practice. All variables included in our analysis were recorded at enrolment or as baseline data in each of the included studies in the PREPARE dataset. All deaths included in this analysis occurred during the hospitalisation from which baseline data were collected.

### Statistical analyses

We restricted our analyses to patients with no missing values for any candidate variables. We calculated the effective sample size required for the development of a new clinical prediction model^21^ based on the inputs R^2^ of <0.05, shrinkage factor of 0.9, 26 parameters and outcome (pneumonia-related mortality) prevalence of 3%. The minimum sample size required was 23 270, with 699 events (events per candidate predictor parameter of at least 26.8).

For the derivation of the PREPARE risk assessment tool, we constructed a multivariable backward regression model, including all candidate variables to assess the strength of the association of each candidate variable on hospitalised pneumonia-related mortality. Associations with 95% CI for adjusted ORs (aORs) that did not cross 1 were considered significant. Then, to determine the weighted points assigned to each candidate variable, we calculated the adjusted log coefficient of each candidate variable from the multivariable model, rounded it to the nearest 0.5 and then doubled the rounded log coefficients to form an integer.^4 7 22 23^ As the PREPARE risk assessment tool is intended to be used by clinicians in settings with all levels of resources, weighted points were assigned to each candidate variable to create a user-friendly risk assessment tool that can be simply calculated without the use of a computer or an application.^24^

To assess the discriminatory ability of the PREPARE risk assessment tool to identify children at risk of hospitalised pneumonia-related mortality, we internally validated the risk assessment tool using bootstrapping methodology with 200 repetitions and calculated the area under the receiver operating characteristic (ROC) (AUC).^21 25 26^ As pulse oximetry is not available in all settings, we conducted a sensitivity analysis of the performance of the PREPARE risk assessment tool excluding pulse oximetry. We used the Hosmer-Lemeshow test to assess the goodness of fit of the PREPARE risk assessment tool by testing the null hypothesis that the fitted values from the model were the same as observed.

We created an ROC curve for the PREPARE risk assessment tool and repeated these analyses without pulse oximetry. We created a risk predictiveness curve to demonstrate the cumulative percentage of children at risk of hospitalised pneumonia-related mortality by predicted risk. We created a calibration plot of the agreement between estimated and observed probabilities for hospitalised pneumonia-related mortality. We conducted decision curve analysis including all candidate variables to estimate the clinical utility of the PREPARE risk assessment tool. We used descriptive statistics to describe characteristics among children who were misclassified (ie, deemed low risk at optimal PREPARE risk assessment scores but died). All analyses were conducted using Stata V.16.1.

## Results

**Figure 1:**
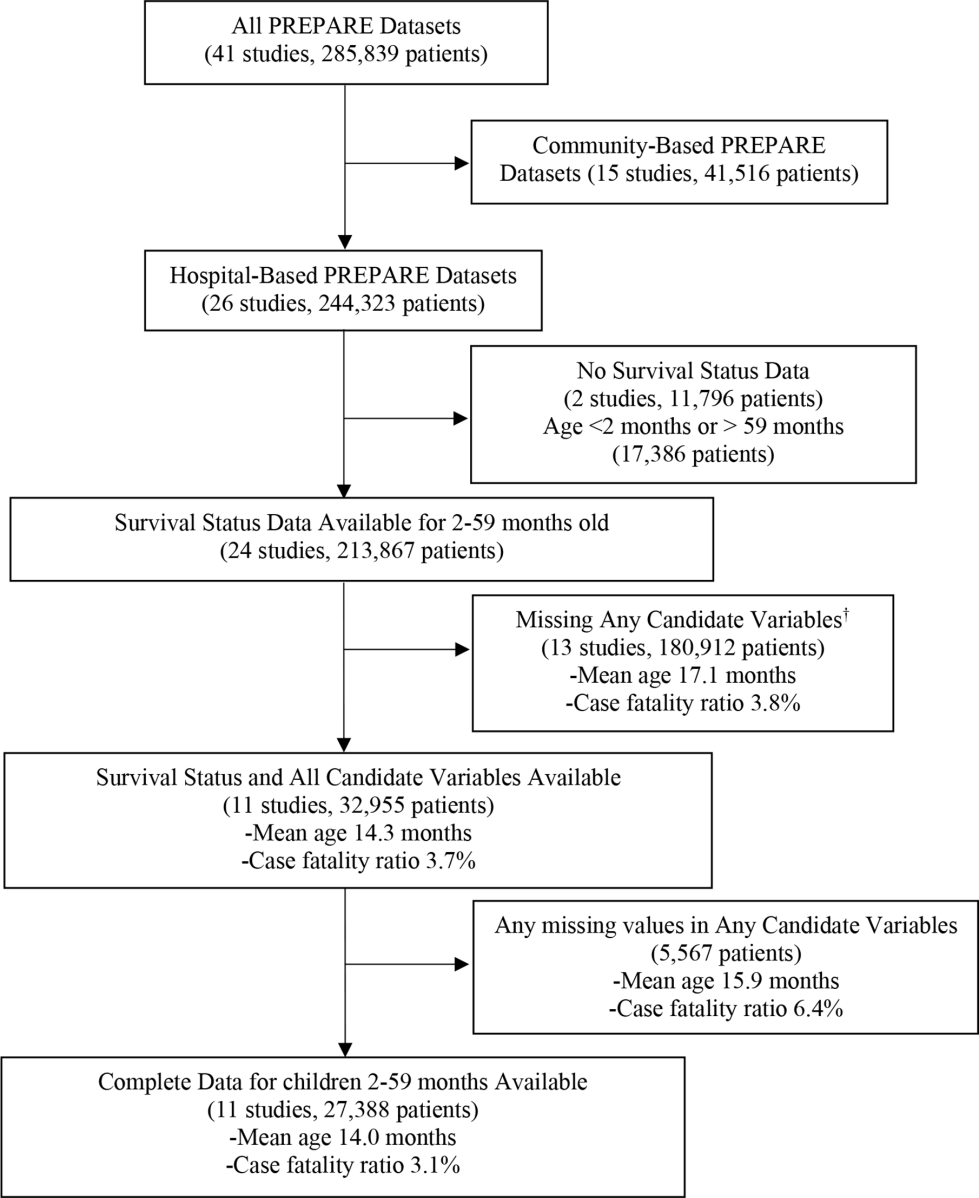
Selection of hospital-based studies included in the derivation and validation of the novel PREPARE risk assessment tool to identify children 2–59 months of age at risk of hospitalised pneumonia-related mortality. ^*^Candidate variables include age, sex, weight-for-age z-score, temperature, respiratory rate, oxygen saturation, presence of chest indrawing, unconsciousness/decreased consciousness, convulsions and cyanosis. PREPARE, Pneumonia Research Partnership to Assess WHO Recommendations.

Of 41 datasets with a total of 285 839 children in the PREPARE dataset, 11 studies with 27 388 children met our inclusion criteria (figure 1). Six of the included studies were randomised controlled trials; two were prospective cohort studies; two were retrospective cohort studies; and one was a prospective case series (table 1). The included studies were conducted in 20 different low-income or middle-income countries in Asia, Africa, Central and South America, the Caribbean and the Middle East. The mean age was 14.0±12.1 months and the case fatality ratio was 3.1%. Children included in the derivation and validation of the PREPARE risk assessment tool were slightly younger than those who did not meet the inclusion criteria (14.0±12.1 months vs 17.1±13.5 months) but did not differ substantially by sex (male 15 862 (57.9%) vs 100 023 (56.0%)), weight-for-age z-score (−1.12±2.00 vs −1.01±1.75) or mortality (n=856 (3.1%) vs n=6877 (3.8%)). Children with any missing parameter for any candidate variable were not included in the derivation or validation of the PREPARE risk assessment tool (online supplemental table 1). The risk predictiveness curve including all children who met the inclusion criteria demonstrated that most children were at low risk for mortality (online supplemental figure 1).

**Table 1:** Characteristics of studies included in the derivation and validation of the PREPARE risk assessment tool

Reference	Study design	Study locations	Age range	Sample size, n	Deaths, n (%)	Inclusion criteria	Exclusion criteria
Addo-Yobo *et al *^43^	Randomised controlled trial	Bogota, Colombia. Kumasi, Ghana. Nagpur, India. Mexico City, Mexico. Durban and Cape Town, South Africa. Ho Chi Minh City, Vietnam. Islamabad, Pakistan Ndola, Zambia	2–59 months	1629	15 (0.9)	Children with WHO-defined pneumonia of any severity*	Children with very severe disease. Children who lived >12 miles from the hospital. Children who had taken one or more antimicrobial drugs for at least 48 hours before presentation. Children who had been admitted to the hospital for any reason within the previous 7 days. Children with oxygen saturation of ≤87% on room air.
Ugpo *et al*^44^	Randomised controlled trial	Bohol, Philippines	6–14 weeks	1102	19 (1.7)	Children who were healthy and lived in the study areas who were to start their routine vaccination	infants who had received the first dose of diptheria, pertussis and tetanus vaccine Acute febrile illness (rectal temperature ≥38°C). Suspected to have neurological disease. History of hospitalisation for and/or treatment for immune suppression.
Basnet *et al*^45^	Randomised controlled trial	Kathmandu, Nepal	2–35 months	638	6 (0.9)	Children with a complaint of cough for <14 days. Children with difficulty breathing for ≤72 hours, presence of lower chest wall indrawing on examination.	Children with recurrent wheezing, heart disease, other severe illness, severe malnutrition, dehydration, haemoglobin <70 g/L, chronic cough, effusion on chest x-ray or history of documented tuberculosis.
Mathew *et al*^46^	Prospective cohort	Chandigarh, India	1–59 months	1868	148 (7.9)	Children with WHO-defined pneumonia of any severity	Duration of illness >7 days. Antibiotics for >24 hours. Previous hospitalisation within the preceding 30 days. Children with wheeze who received a single dose of bronchodilator and whose symptoms disappeared.
WS Clara, unpublished data 2012^47^	Retrospective cohort	Chiriqui Province, Panama	0–59 months	57	1 (1.7)	Children with severe acute respiratory infection case definition	None
Bénet *et al*^48^	Prospective case control	Phnom Penh, Cambodia. Beijing, China. Port au Prince, Haiti. Lucknow, India. Pune-Vadu, India. Antananarivo, Madagascar. Bamako, Mali. Ulaanbaatar, Mongolia. San Lorenzo, Paraguay.	2–59 months	855	19 (2.2)	Children with WHO-defined pneumonia with first symptoms lasting <14 days. Children with radiographic pneumonia (WHO criteria).	Children with wheezing at auscultation. Children whose parent or legal guardian declined to sign the informed consent statement.
McCollum *et al*^49^	Prospective cohort	Mchinji and Lilongwe Districts, Malawi	0–59 months	14 681	460 (3.1)	Children with WHO-defined pneumonia of any severity	None
Wulandari *et al*^50^	Retrospective cohort	West Java, Indonesia	0–59 months	1125	62 (5.5)	Children with WHO-defined pneumonia	Children aged >5 years. Children with hospital-acquired pneumonia.
Klugman *et al*^51^	Randomised controlled trial	Johannesburg, South Africa	0–59 months	9668	427 (4.4)	ICD-10 codes for pneumonia	Children with progressive underlying neurological disorder, history of seizures or infantile spasms, or a low likelihood of receiving three doses of vaccine.
Asghar *et al*^52^	Randomised controlled trial	Dhaka, Bangladesh. Guayaquil, Ecuador. Chandigarh, India. Mexico City, Mexico. Rawalpindi, Pakistan. Multan, Pakistan. Sana’a, Yemen. Lusaka, Zambia.	2–59 months	894	46 (5.1)	Children with WHO-defined very severe pneumonia	Children with wheezing, a history of three or more attacks or known asthma. Known heart disease. Duration of illness >10 days. History of serious adverse reaction to any study drug. Admission to the hospital for >24 hours within the past 7 days. Documented evidence of injectable antibiotic treatment for >24 hours before enrolment. Stridor, known renal failure or not passed urine during the past 6 hours, evidence of cerebral malaria, evidence of bacterial meningitis, clinical jaundice. Residence of patient in an area where follow-up was not possible. Empyema or presence of pneumatoceles on chest radiograph.
Wadhwa *et al*^53^	Randomised controlled trial	New Delhi, India	2–24 months	438	7 (1.6)	Breathing >50 breaths/min in children 2–12 months and >40 breaths/min in children >13–24 months. Crepitations on auscultation. Presence of chest indrawing. Very severe pneumonia: severe pneumonia (with or without chest indrawing) and any general danger sign (ie, lethargy or inability to drink or convulsions) or central cyanosis.	Need for mechanical ventilation or inotropic medications. Major congenital anomalies. Inborn errors of metabolism. Chronic disorders such as renal failure, pre-existing seizure disorders, surgical or other conditions that interfered with oral feeding, HIV infection. Born to mothers with documented HIV infection. Active measles. Severe malnutrition that required separate medical attention. Any other serious underlying medical condition.

* WHO-defined pneumonia: presence of age-specific fast breathing, lower chest indrawing, or general danger signs in children with a cough or difficulty breathing. Severe pneumonia: cough and/or difficulty breathing, and central cyanosis or inability to drink. Very severe pneumonia: cough or difficulty breathing with one or more danger signs—convulsions, drowsiness (altered consciousness), inability to drink, severe clinical malnutrition and stridor at rest.^14^

PREPARE, Pneumonia Research Partnership to Assess WHO Recommendations.

### Derivation of the PREPARE risk assessment tool

Weight-for-age z-score of <−3 (aOR 5.16, 95% CI 4.37 to 6.09), body temperature of <35.5°C (aOR 4.80, 95% CI 3.05 to 5.57) and SpO_2_of <90% (aOR 2.99, 95% CI 2.51 to 3.58) were most strongly associated with hospitalised pneumonia-related mortality among all included children (table 2). The PREPARE risk assessment tool had a score ranging from 0 to 17 and incorporated patient age, sex, weight-for-age z-score, body temperature, respiratory rate, unconsciousness or decreased level of consciousness, convulsions, cyanosis and hypoxaemia at baseline (table 3).

**Table 2:** Multivariable regression model for hospitalised pneumonia-related mortality among all included children aged 2–59 months (n=27 388)

Factor	Survived, n (%)	Died, n (%)	OR	95% CI	Adjusted OR	95% CI
All*	26 532 (96.9)	856 (3.1)	**–**	**–**	**–**	**–**
Age category						
12–59 months	10 927 (98.2)	201 (1.8)	Referent	–	Referent	–
6–11 months	7064 (96.9)	222 (3.1)	1.71	(1.41 to 2.07)	1.68	(1.37 to 2.06)
2–5 months	8541 (95.2)	433 (4.8)	2.76	(2.32 to 3.27)	2.35	(1.96 to 2.82)
Sex						
Male	15 412 (97.2)	450 (2.8)	Referent	–	Referent	–
Female	11 120 (96.5)	198 (3.5)	1.25	(1.09 to 1.43)	1.36	(1.18 to 1.57)
Weight-for-age z-score						
>−2	19 869 (98.5)	302 (1.5)	Referent	–	Referent	–
−2 to −3	3357 (94.4)	172 (4.9)	3.37	(2.78 to 4.087)	2.72	(2.23 to 3.31)
<−3	3306 (89.6)	382 (10.4)	7.60	(6.51 to 8.88)	5.16	(4.37 to 6.09)
Body temperature category						
<35.5°C	205 (87.6%)	29 (12.4%)	4.68	(3.14 to 6.97)	4.80	(3.05 to 7.57)
35.5°C to 37.9°C	18 122 (97.1)	548 (2.9)	Referent	–	Referent	–
>38°C	8205 (96.7)	279 (3.3)	1.12	(0.97 to 1.30)	1.04	(0.89 to 1.22)
Respiratory rate (breaths/min)						
≤Age-specific cut-off*	5345 (98.0)	107 (2.0)	Referent	–	Referent	–
0–9 above age-specific cut-off*	8029 (97.5)	205 (2.5)	1.27	(1.01 to 1.61)	1.20	(0.93 to 1.55)
10–19 beats/min above age-specific cut-off*	7882 (97.2)	225 (2.8)	1.42	(1.13 to 1.80)	1.03	(0.79 to 1.33)
>20 above age-specific cut-off*	5276 (94.3)	319 (5.7)	3.02	(2.41 to 3.77)	1.72	(1.32 to 2.23)
Lower chest indrawing						
No	8346 (98.0)	167 (2.0)	Referent	–	Referent	–
Yes	18 186 (96.4)	689 (3.6)	1.89	(1.60 to 2.25)	1.26	(1.00 to 1.48)
Unconscious/decreased consciousness						
No	25 587 (97.1)	760 (2.9)	Referent	–	Referent	–
Yes	945 (90.8)	96 (9.2)	3.42	(2.74 to 4.27)	1.91	(1.49 to 2.44)
Convulsions						
No	25 100 (97.0)	786 (3.0)	Referent	–	Referent	–
Yes	1432 (95.3)	70 (4.7)	1.56	(1.22 to 2.00)	2.87	(2.16 to 3.81)
Cyanosis						
No	25 628 (97.4)	679 (2.6)	Referent	–	Referent	–
Yes	904 (83.6)	177 (16.4)	7.39	(6.18 to 8.83)	2.34	(1.90 to 2.88)
Oxygen saturation category						
<90%	4318 (90.4)	457 (9.6)	6.41	(5.52 to 7.45)	2.99	(2.51 to3.58)
90%–92%	4342 (97.7)	104 (2.3)	1.45	(1.16 to 1.82)	1.23	(0.98 to 1.55)
93%–100%	17 872 (98.4)	295 (1.6)	Referent	–	Referent	–

* ≥50 breaths/min for children 2–11 months old or ≥40 breaths/min for children 12–59 months old.

**Table 3:** Components of the PREPARE risk assessment tool including all children who met the inclusion criteria (n=27 388)

Factor	Adjusted log coefficient	PREPARE score*
Age category		
12–59 months	–	–
6–11 months	0.52	+1
2–5 months	0.85	+2
Sex		
Male	–	–
Female	0.31	+1
Weight-for-age z-score		
>−2	–	–
−2 to −3	1.00	+2
<−3	1.64	+3
Body temperature category		
<35.5°C	1.57	+3
35.5°C to 37.9°C	–	–
>38°C	0.04	+0
Respiratory rate (breaths/min)		
≤Age-specific cut-off†	–	–
0–9 above age-specific cut-off†	0.18	+0
10–19 beats/min above age-specific cut-off†	0.03	+0
≥20 above age-specific cut-off†	0.54	+1
Lower chest indrawing		
No	–	–
Yes	0.19	+0
Unconscious/decreased consciousness		
No	–	–
Yes	0.65	+1
Convulsions		
No	–	–
Yes	1.05	+2
Cyanosis		
No	–	–
Yes	0.85	+2
Oxygen saturation category		
<90%	1.10	+2
90%–92%	0.21	+0
93%–100%	–	–

* To determine the weighted points assigned to each candidate variable from the multivariable model, we calculated the adjusted log coefficient of each candidate variable, rounded it to the nearest 0.5 and then doubled the rounded log coefficients to form an integer.

† ≥50 breaths/min for children 2–11 months old or ≥40 breaths/min for children 12–59 months old.

PREPARE, Pneumonia Research Partnership to Assess WHO Recommendations.

Our sensitivity analysis excluding pulse oximetry demonstrated a weight-for-age z-score of <−3, body temperature of <35.5°C and cyanosis were most strongly associated with hospitalised pneumonia-related mortality among all children (online supplemental table 2). The PREPARE risk assessment tool including all children and excluding pulse oximetry had a score ranging from 0 to 20 (online supplemental table 3).

### Validation of the PREPARE risk assessment tool

**Figure 2:**
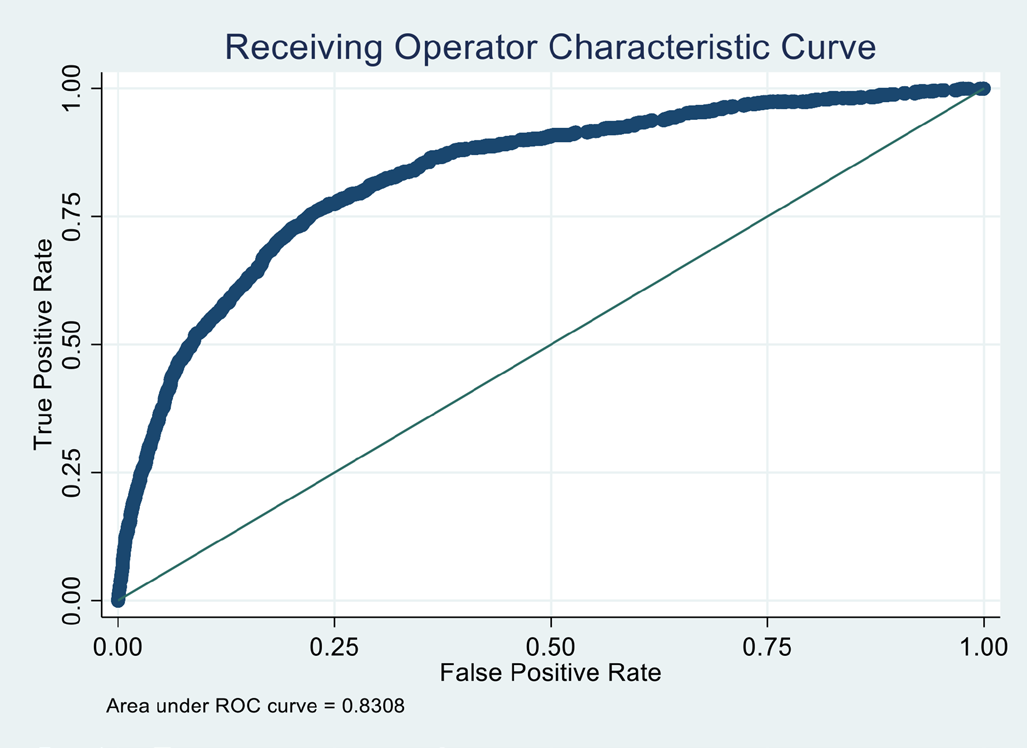
Receiver operating characteristic curve for the PREPARE risk assessment tool for children at risk of hospitalised pneumonia-related mortality among children 2–59 months of age (n=27 388). Area under receiver operating curve: 0.83 (95% CI 0.81 to 0.84). PREPARE, Pneumonia Research Partnership to Assess WHO Recommendations.

The internal validation of the PREPARE risk assessment tool using the bootstrap method demonstrated an AUC of 0.83 (95% CI 0.81 to 0.84) among all children (figure 2) and 0.81 (95% CI 0.79 to 0.82) when excluding pulse oximetry. The PREPARE risk assessment tool had maximised sensitivity with concurrent maximised specificity at a score of ≥5 (72.6% sensitivity, 76.5% specificity; +likelihood ratio [LR] of 3.09 (95% CI 2.89 to 3.30) and −LR of 0.36 (95% CI 0.31 to 0.42)) in identifying children at risk of hospitalised pneumonia-related mortality. A score of ≥4 had 81.8% sensitivity, 65.4% specificity, +LR of 2.37 (95% CI 2.25 to 2.49) and −LR of 0.28 (95% CI 0.23 to 0.34), and a score of ≥6 demonstrated 61.3% sensitivity and 84.4% specificity with a +LR of 3.93 (95% CI 3.61 to 4.29) and a −LR of −0.46 (95% CI 0.41 to 0.52).

### Goodness of fit, calibration and decision curve analysis

The Hosmer-Lemeshow test to assess the goodness-of-fit demonstrated a p value of <0.001, meaning that the observed and expected proportions were not the same across all groups. The calibration plot of observed against expected probabilities for the assessment of prediction model performance demonstrated reasonably good model calibration (online supplemental figure 2). The PREPARE risk assessment tool had higher net benefit than individual candidate variables in predicting pneumonia-related mortality (online supplemental figure 3).

### Misclassified patients in validation of the PREPARE risk assessment tool

At a PREPARE risk assessment tool score of <4, 75 children (16.9% of all deaths) were classified as low risk but died (online supplemental table 4). At a score of <5, 113 children died (25.5% of all deaths) and were misclassified for their risk of hospitalised pneumonia-related mortality. At a score of <6, 160 children died (36.1% of all deaths) and were incorrectly classified for their risk of hospitalised pneumonia-related mortality. Misclassified children commonly had a weight-for-age z-score of ≥2, normothermia and lower chest indrawing; were of normal consciousness; and were not cyanotic. No children with hypothermia were misclassified for their risk of hospitalised pneumonia-related mortality at a score of <4, <5 or <6.

## Discussion

Early identification of children at risk of mortality during hospitalisation may allow for the allocation of resources to potentially prevent such deaths.^27^ The PREPARE risk assessment tool was derived from the largest and most geographically diverse patient population of all existing risk assessment tools for hospitalised pneumonia-related mortality and included variables that are routinely assessed in clinical practice. Our novel risk assessment tool demonstrated good discriminatory ability when internally applied to patients from a range of settings both with and without use of pulse oximetry. After external validation, the PREPARE risk assessment tool may be used to identify children at risk of hospitalised pneumonia-related mortality and could be used for monitoring of children hospitalised with pneumonia.

The PREPARE risk assessment tool includes the assessment of a patient’s age, sex, weight-for-age z-score, body temperature, respiratory rate, level of consciousness, presence of convulsions, cyanosis and SpO_2_. Weight-for-age z-score, level of consciousness and SpO_2_ of <90% were associated with mortality in the RISC-Malawi and PERCH scores.^6 7^ Younger age was also associated with mortality in the PERCH score.^7^ We additionally identify findings of hypothermia (ie, <35.5°C), tachypnoea and convulsions associated with mortality among children hospitalised with pneumonia. Hypothermia was strongly associated with mortality, second only to malnutrition. No children with hypothermia were misclassified for their risk of hospitalised pneumonia-related mortality at PREPARE risk assessment tool scores of <4, <5 or <6. Most variables included in the PREPARE risk assessment tool can be easily assessed by providers of many training levels in various settings. However, the assessment of SpO_2_ requires a pulse oximeter with paediatric probes and additional training in the accurate use of pulse oximetry. Prior studies demonstrate that community level health workers and first-level health facility workers can be trained to accurately use pulse oximetry.^28 29^ The PREPARE risk assessment tool also had good discriminatory value in our sensitivity analysis excluding pulse oximetry. Therefore, the PREPARE risk assessment tool may be useful in identifying children at risk of hospitalised pneumonia-related mortality in settings with limited access to pulse oximetry.

Due to missing data in the included datasets, we were not able to assess if the presence of wheezing was potentially protective. Wheezing has been associated with lower mortality rates among children,^30^ is more common in viral pneumonia and bronchiolitis than bacterial pneumonia,^31^,^33^ and serves as a protective variable in other clinical prediction models.^4 6^ However, accurate auscultation requires a stethoscope and experienced clinicians. Moreover, prior studies demonstrate variable inter-rater reliability for the detection of wheezing in children.^34^,^36^ Thus, the exclusion of wheezing from the PREPARE risk assessment tool may allow providers in various settings and with varying training levels to more easily use this risk assessment tool.

The WHO Integrated Management of Childhood Illness guidelines recommend that HIV-uninfected children 2–59 months of age who have lower chest indrawing and no danger signs be treated with oral amoxicillin at home without hospital referral.^12^ This recommendation has been criticised with some calling for its revision.^37 38^ The presence of lower chest indrawing was included in both the RISC and modified Respiratory Index of Severity in Children (mRISC), but not in the RISC-Malawi or PERCH risk assessment tools for hospitalised pneumonia-related mortality.^4^,^7^ Lower chest indrawing was not associated with mortality in our risk assessment tool. The RISC and mRISC scores demonstrated an association between the presence of chest indrawing and mortality.^4 5^ Accordingly, the PREPARE risk assessment tool should be externally validated prior to implementation with careful attention paid to the presence of lower chest indrawing, given the differing findings in prior models.

Our novel risk assessment tool demonstrated good discriminatory ability in this within-sample validation in children from 20 low-income and middle-income countries in Asia, Africa, Central and South America, the Caribbean and the Middle East. Prior clinical prediction models developed in single countries (ie, RISC, mRISC and RISC-Malawi) have demonstrated AUC from 0.80 to 0.92 when internally validated,^4^,^6^ while the PERCH score, derived from five countries in sub-Saharan Africa as well as Thailand and Bangladesh, had an AUC of 0.76 on internal validation.^7^ However, when externally applied to patients in various settings, only the RISC-Malawi score had fair discriminatory ability.^9^ The PREPARE risk assessment tool transcends region-specific issues, such as differing epidemiology of causative viruses or bacteria and variations in availability of measures such as chest radiography, supplemental oxygen, antibiotic availability and unmeasured variables that can contribute to morbidity, given its derivation and validation from a widely representative patient population. However, the PREPARE risk assessment tool must be externally validated prior to implementation. Though the PREPARE risk assessment tool had maximised test characteristics at a score of ≥5, some patients were misclassified for their risk of hospitalised pneumonia-related mortality at that score. Higher scores had fewer misclassified children but in turn had lower sensitivity. Thus, clinicians may use higher PREPARE risk assessment tool scores to identify children at high risk of hospitalised pneumonia-related mortality, but scores above 5 should not be reliably used to rule out the possibility of hospitalised pneumonia-related mortality. Future studies assessing the impact of the implementation of the PREPARE risk assessment tool must be compared with routine clinical care.

### Limitations

Our study is subject to several limitations. As many studies in the PREPARE dataset used tachypnoea as an entry point sign to diagnose pneumonia, tachypnoea as a predictor of mortality may have been overestimated. However, we assessed the contribution of varying severity of tachypnoea to mortality, which may reduce potential bias introduced by including tachypnoea as a predictor. Furthermore, the WHO manual on oxygen therapy for children recommends oxygen therapy for children with severe lower chest wall indrawing, respiratory rate of ≥70 breaths/min and head nodding in settings where pulse oximetry is not available.^20^ As we conducted an analysis of previously collected data, not all candidate variables for the novel risk assessment tool were available in large numbers in the PREPARE dataset. Specifically, we were not able to assess HIV infection status as a candidate variable, which has been associated with mortality in other studies.^4 39 40^ Future studies incorporating HIV infection status into the PREPARE risk assessment tool in endemic regions may be needed. Furthermore, given the retrospective nature of this study, we were not able to assess several clinical variables that have been associated with mortality in other studies, such as grunting, duration of illness^7^ or signs such as apnoea, gasping, nasal flaring or head nodding,^14^ or anaemia and pallor.^41 42^ Apnoea, gasping, nasal flaring and head nodding have not been incorporated in other risk assessment tools.^4^,^7^ Future studies incorporating these signs into risk assessment tools may be warranted. The case fatality rate was higher among children with missing data than among those who had complete data. This may have been due to deaths that occurred early in the hospitalisation before time was granted to fully collect clinical data. Additionally, six of the datasets included in our analysis came from randomised controlled trials, which tend to be highly selective of patients and may explain part of the lower case fatality rate in our derivation and validation populations compared with children who were excluded from our analysis. An additional limitation is the possibility of confounding by disease severity and management variation across regions. For example, some variables included in the PREPARE risk assessment tool, such as SpO_2_, may be modified through interventions (eg, the use of supplemental oxygen). Lastly, our analysis did not assess the role of the quality of care, supplemental oxygen availability, regional variations in clinical care or antibiotics administered.

## Conclusions

The PREPARE risk assessment tool is a novel tool that had good discriminatory ability at hospital admission to identify children at risk of hospitalised pneumonia-related mortality when applied to >27 000 children in 20 low-income and middle-income countries. The PREPARE risk assessment tool may help direct resources to children at highest risk of hospitalised pneumonia-related mortality in resource-limited settings. This novel tool includes routinely collected variables that can be assessed by healthcare providers of all levels. External validation of the PREPARE risk assessment tool and a comparison of its impact on hospitalised pneumonia-related mortality among children across various settings compared with standard clinical care may be warranted.

## Supplementary material

10.1136/bmjgh-2021-008143 online supplemental file 1

## Data Availability

Data are available upon reasonable request.
