# Supplementary material for: Derivation and validation of a novel risk assessment tool to identify children aged 2–59 months at risk of hospitalised pneumonia-related mortality in 20 countries
Source: BMJ Glob Health. 2022 Apr 15;7(4):e008143. doi: 10.1136/bmjgh-2021-008143 (PMC9014031; doi:10.1136/bmjgh-2021-008143)

Supplemental Table 1. Proportion of children with survival status available but had at least one missing parameter included in the PREPARE* risk assessment tool

	**Missing Parameter, N=32,955, n (%)**
Age	0 (0.0%)
Sex	537 (1.6%)
Weight-for-Age Z Score	1,988 (6.0%)
Body temperature category	1,748 (5.3%)
Respiratory rate (breaths/min)	2,806 (8.5%)
Lower chest indrawing	153 (0.5%)
Unconscious/decreased consciousness	180 (0.5%)
Convulsions	359 (1.1%)
Cyanosis	156 (0.5%)
Oxygen saturation category	3,004 (9.1%)

*Pneumonia REsearch Partnership to Assess WHO Recommendations

Supplemental Table 2. Multivariable regression model for hospitalized pneumonia-related mortality among all children aged 2-59 months excluding pulse oximetry (n=27,388)

Factor	Survived, n (%)	Died, n (%)	Odds Ratio	95% Confidence Interval	Adjusted Odds Ratio	95% Confidence Interval
**All***	26,532 (96.9%)	856 (3.1%)	--	--	--	--
**Age category**						
12-59 months	10,927 (98.2%)	201 (1.8%)	*Referent*	--	*Referent*	--
6-11 months	7,064 (96.9%)	222 (3.1%)	1.71	(1.41, 2.07)	1.82	(1.48, 2.23)
2-5 months	8,541 (95.2%)	433 (4.8%)	2.76	(2.32, 3.27)	2.58	(2.15, 3.09)
**Sex**						
Male	15,412 (97.2%)	450 (2.8%)	*Referent*	--	*Referent*	--
Female	11,120 (96.5%)	198 (3.5%)	1.25	(1.09, 1.43)	1.38	(1.19, 1.59)
**Weight-for-Age Z Score**						
≥-2	19,869 (98.5%)	302 (1.5%)	*Referent*	--	*Referent*	--
-2 to -3	3,357 (94.4%)	172 (4.9%)	3.37	(2.78, 4.087)	2.91	(2.39, 3.55)
<-3	3,306 (89.6%)	382 (10.4%)	7.60	(6.51, 8.88)	5.86	(5.00, 6.91)
**Body temperature category**						
<35.5^{0}C	205 (87.6%)	29 (12.4%)	4.68	(3.14, 6.97)	4.82	(3.10, 7.51)
35.5^{0}C to 37.9^{0}C	18,122 (97.1%)	548 (2.9%)	*Referent*	--	*Referent*	--
≥38^{0}C	8,205 (96.7%)	279 (3.3%)	1.12	(0.97, 1.30)	1.06	(0.91, 1.24)
**Respiratory rate (breaths/min)**						
≤ age-specific cutoff[†]	5,345 (98.0%)	107 (2.0%)	*Referent*	--	*Referent*	--
0-9 above age-specific cutoff[†]	8,029 (97.5%)	205 (2.5%)	1.27	(1.01, 1.61)	1.27	(0.99, 1.64)
10-19 bpm above age-specific cutoff[†]	7,882 (97.2%)	225 (2.8%)	1.42	(1.13, 1.80)	1.18	(0.91, 1.53)
≥20 above age-specific cutoff[†]	5,276 (94.3%)	319 (5.7%)	3.02	(2.41, 3.77)	2.24	(1.74, 2.90)
**Lower chest indrawing**						
No	8,346 (98.0%)	167 (2.0%)	*Referent*	--	*Referent*	--
Yes	18,186 (96.4%)	689 (3.6%)	1.89	(1.60, 2.25)	1.43	(1.18, 1.75)
**Unconscious/decreased consciousness**						
No	25,587 (97.1%)	760 (2.9%)	*Referent*	--	*Referent*	--
Yes	945 (90.8%)	96 (9.2%)	3.42	(2.74, 4.27)	2.14	(1.67, 2.73)
**Convulsions**						
No	25,100 (97.0%)	786 (3.0%)	*Referent*	--	*Referent*	--
Yes	1,432 (95.3%)	70 (4.7%)	1.56	(1.22, 2.00)	2.81	(2.12, 3.72)
**Cyanosis**						
No	25,628 (97.4%)	679 (2.6%)	*Referent*	--	*Referent*	--
Yes	904 (83.6%)	177 (16.4%)	7.39	(6.18, 8.83)	3.56	(2.92, 4.35)

[†]≥50 breaths/minute for children 2-11 months old, or ≥40 breaths/minute for children 12-59 months old.

Supplemental Table 3. Components of the PREPARE* Risk Assessment Tool among all children but excluding pulse oximetry (n=27,388)

**Factor**	**Adjusted log coefficient**	**PREPARE Score**†
**Age category**		
12-59 months	--	--
6-11 months	0.60	+1
2-5 months	0.95	+2
**Sex**		
Male	--	--
Female	0.32	+1
**Weight-for-Age Z Score**		
>-2	--	--
-2 to -3	1.07	+2
<-3	1.77	+4
**Body temperature category**		
<35.5^{0}C	1.57	+3
35.5^{0}C to 37.9^{0}C	--	--
≥38^{0}C	0.06	+0
**Respiratory rate (breaths/min)**		
≤ age-specific cutoff‡	--	--
0-9 above age-specific cutoff‡	0.24	+0
10-19 bpm above age-specific cutoff‡	0.16	+0
≥20 above age-specific cutoff‡	0.80	+2
**Lower chest indrawing**		
No	--	--
Yes	0.36	+1
**Unconscious/decreased consciousness**		
No	--	--
Yes	0.76	+2
**Convulsions**		
No	--	--
Yes	1.03	+2
**Cyanosis**		
No	--	--
Yes	1.27	+3

*Pneumonia REsearch Partnership to Assess WHO Recommendations

†To determine the weighted points assigned to each candidate variable from the multivariable model, we calculated the adjusted log coefficient of each candidate variable, rounded it to the nearest 0.5 and then doubled the rounded log coefficients to form an integer.

‡≥50 breaths/minute for children 2-11 months old, or ≥40 breaths/minute for children 12-59 months old.

Supplemental Table 4. Characteristics of children misclassified by optimal scores in the PREPARE* risk assessment tool.

	Children Misclassified at a Score of <4 (N=75), n (%)	Children Misclassified at a Score of <5 (N=113), n (%)	Children Misclassified at a Score of <6 (N=160), n (%)
**Age category**			
12-59 months	31 (41.3%)	42 (37.2%)	57 (35.6%)
6-11 months	24 (32.0%)	36 (31.8%)	49 (30.6%)
2-5 months	20 (26.7%)	35 (31.0%)	54 (33.8%)
**Sex**			
Male	30 (40.0%)	55 (48.7%)	76 (47.5%)
Female	45 (60.0%)	58 (51.3%)	84 (52.5%)
**Weight-for-Age Z Score**			
≥-2	70 (93.3%)	94 (83.2%)	115 (71.9%)
-2 to -3	5 (6.7%)	13 (11.5%)	27 (16.9%)
<-3	--	6 (5.3%)	18 (11.2%)
**Body temperature category**			
$<35.5^0$C	--	--	--
35.5^0C to 37.9^0C	56 (74.7%)	76 (67.3%)	110 (68.7%)
$\geq 38^0$C	19 (25.3%)	37 (32.7%)	50 (31.3%)
**Respiratory rate (breaths/min)**			
≤ age-specific cutoff[†]	10 (13.3%)	16 (14.2%)	25 (15.6%)
0-9 above age-specific cutoff[†]	30 (40.0%)	36 (31.9%)	45 (28.1%)
10-19 bpm above age-specific cutoff[†]	23 (30.7%)	38 (33.6%)	53 (33.1%)
≥20 above age-specific cutoff[†]	12 (16.0%)	23 (20.3%)	37 (23.2%)
**Lower chest indrawing**			
No	23 (30.7%)	38 (33.6%)	52 (32.5%)
Yes	52 (69.3%)	75 (66.4%)	108 (67.5%)
**Unconscious/decreased consciousness**			
No	71 (94.7%)	107 (94.7%)	149 (93.1%)
Yes	4 (5.3%)	6 (5.3%)	11 (6.9%)
**Convulsions**			
No	75 (100.0%)	111 (98.2%)	154 (96.3%)
Yes	--	2 (1.8%)	6 (3.7%)
**Cyanosis**			
No	73 (97.3%)	109 (96.5%)	153 (95.6%)
Yes	2 (2.7%)	4 (3.5%)	7 (4.4%)
**Oxygen saturation category**			
<90%	3 (4.0%)	15 (13.3%)	34 (21.2%)
90-92%	21 (28.0%)	25 (22.1%)	31 (19.4%)
93-100%	51 (68.0%)	73 (64.6%)	95 (59.4%)

*Pneumonia REsearch Partnership to Assess WHO Recommendations

[†]≥50 breaths/minute for children 2-11 months old, or ≥40 breaths/minute for children 12-59 months old.

Supplemental Table 5. Author reflexivity statement

**1. How does this study address local research and policy priorities?**	This study furthers clinicians' ability to recognize children at-risk of pneumonia-related mortality in varied low- and middle-income countries. Pneumonia is the leading cause of death among infants and children aged 1-59 months in low- and middle-income countries.
**2. How were local researchers involved in study design?**	Local researchers helped conceptualize the studies included in this analysis. They were involved in the study design, data collection, data management, results interpretation, and revisions of the submitted manuscript.
**3. How has funding been used to support the local research team?**	Funding from this project brought local investigators from the included sites together for a meeting to create the PREPARE dataset and provided support for the data management and consolidation performed by local investigators.
**4. How are research staff who conducted data collection acknowledged?**	Research staff who conducted data collection are included as either authors or in the acknowledgements in the primary papers from which these data were obtained.
**5. Do all members of the research partnership have access to study data?**	All members of the research partnership may access the data through requests to Dr. Yasir Nisar.
**6. How was data used to develop analytical skills within the partnership?**	All partners had input on the analytic approach. All feedback was incorporated into the analytic approach through multiple revisions.
**7. How have research partners collaborated in interpreting study data?**	All partners were involved in the interpretation of the results through review of multiple drafts of the primary studies as well as the manuscript included here.
**8. How were research partners supported to develop writing skills?**	All partners contributed to the critical review and revision of this manuscript.
**9. How will research products be shared to address local needs?**	If the presented clinical prediction rule performs well in external validation, it may be shared with all local partners to consider for possible implementation.
**10. How is the leadership, contribution, and ownership of this work by LMIC researchers recognized within the authorship?**	More than 60% of the authors are affiliated with low- and middle-income countries in this manuscript. We acknowledge that, despite the data used in this analysis originating in low- and middle-income countries, the authorship team has significant representation from high-income countries. This is due to longstanding partnerships that led to the availability of these data.
**11. How have early career researchers across the partnership been included within the authorship team?**	We have included early career researchers (CAR, SH) in the authorship team. The remainder of the authors are mid-career to senior-level investigators. We acknowledge that both early career researchers are based in a high-income country.
**12. How has gender balance been addressed within the authorship?**	Eighteen of the authors are female and the remainder are male.
**13. How has the project contributed to training of LMIC researchers?**	Though the majority of investigators in this study are mid-career to senior-level, junior investigators in low- and middle-income countries were involved in the primary studies.
**14. How has the project contributed to improvements in local infrastructure?**	This project has not directly contributed to improvements in local infrastructure.
**15. What safeguarding procedures were used to protect local study participants and researchers?**	There was no primary data collection as part of this project, therefore this question is not directly applicable. Local study participants were safeguarded under the design of each individual study from which the pooled data for this analysis came.

Supplemental Figure 1. Risk predictiveness curve for the PREPARE* risk assessment tool including all children

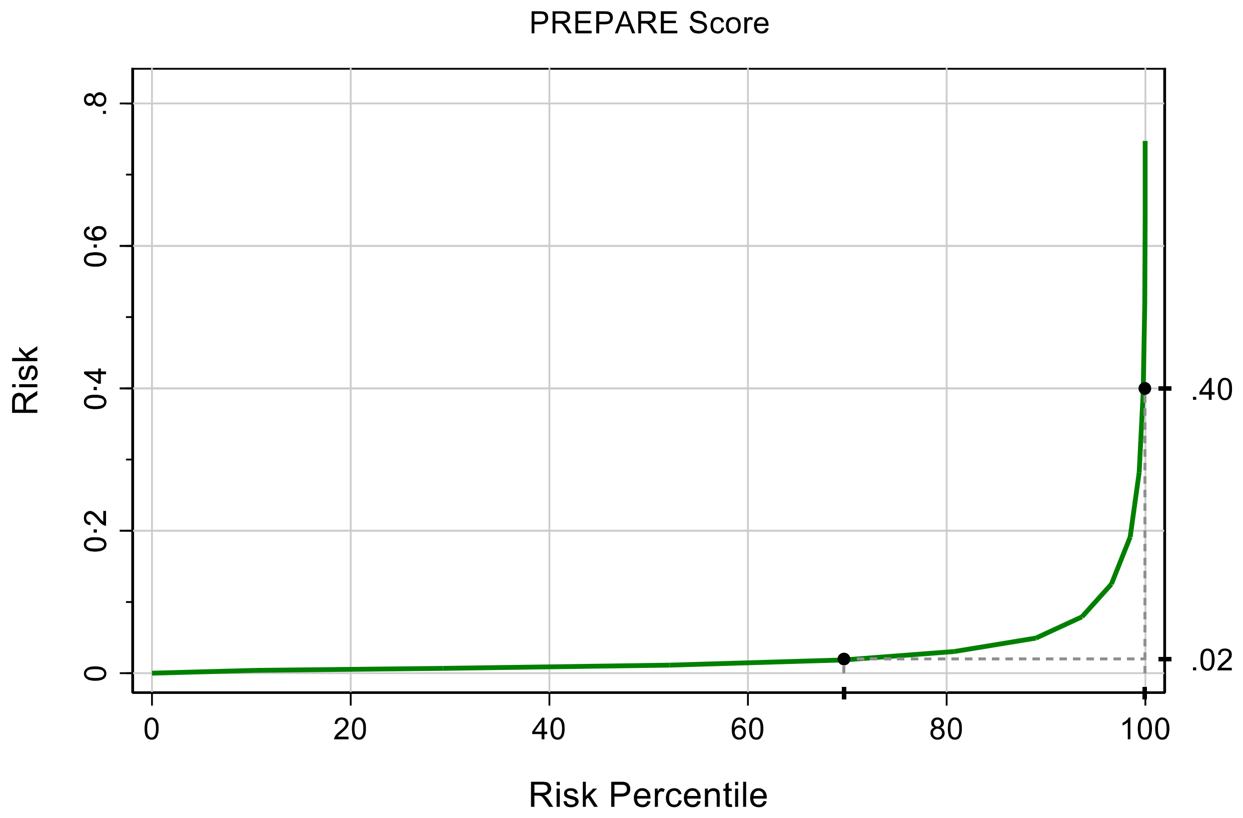

Supplemental Figure 2. Calibration plot for the PREPARE* risk assessment tool for children at risk of hospitalized pneumonia-related mortality among children 2-59 months of age

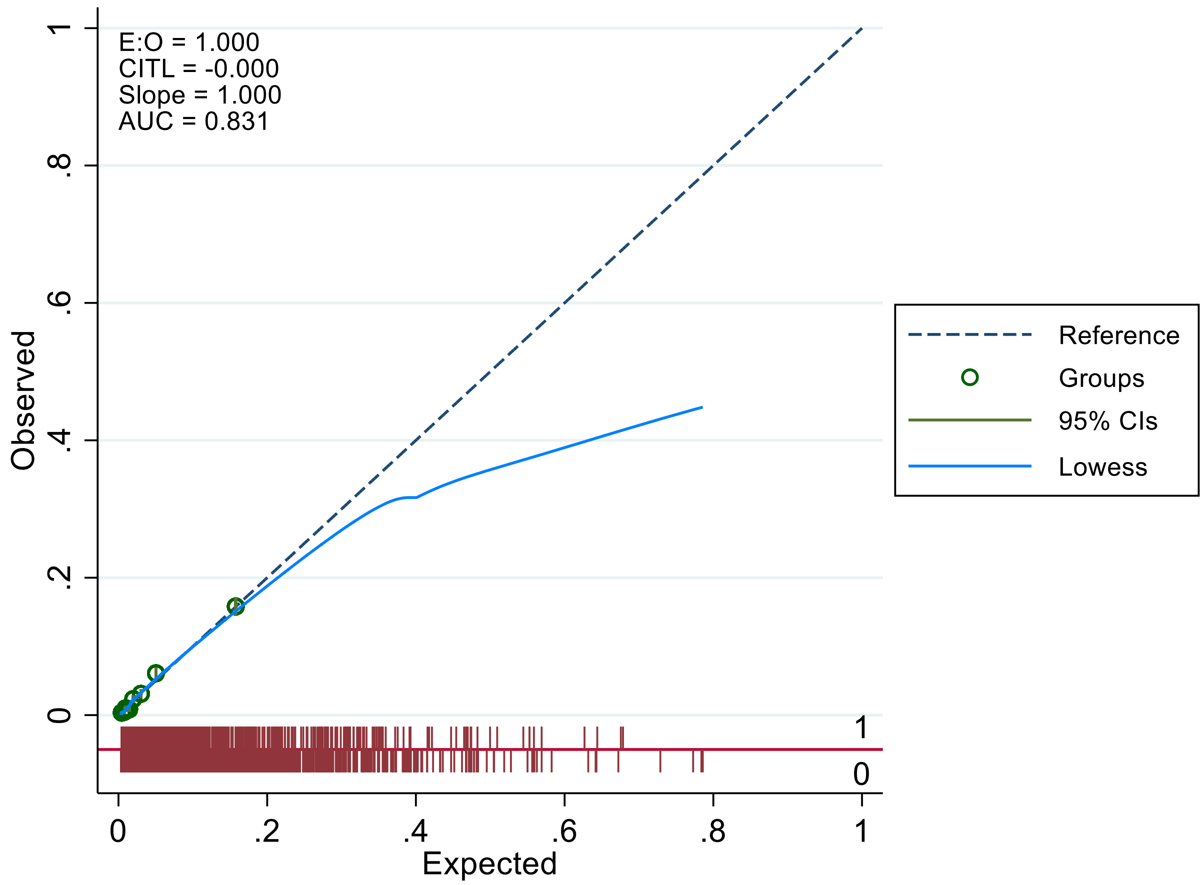

*Pneumonia REsearch Partnership to Assess WHO Recommendations

Supplemental Figure 3. Decision curve analysis of the PREPARE* risk assessment tool

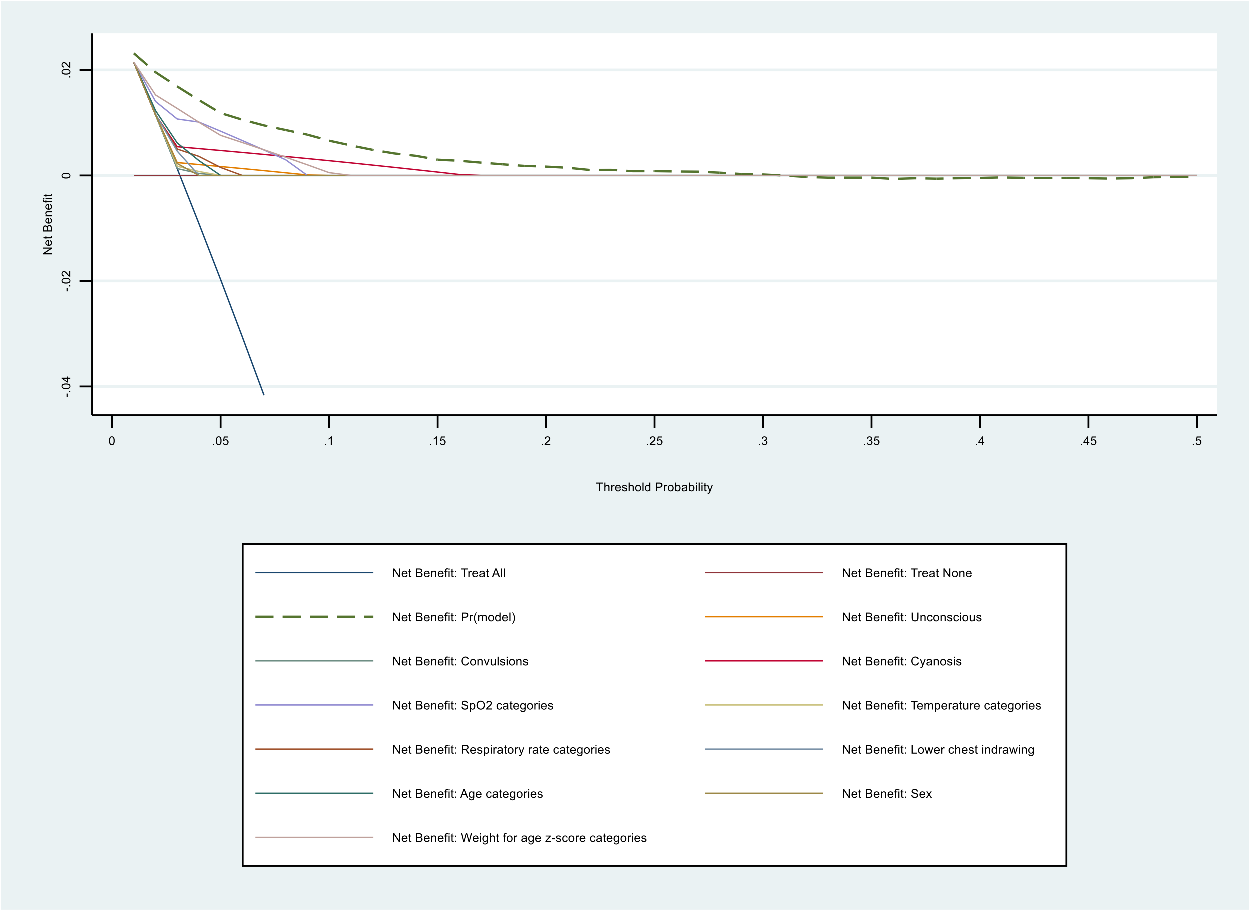

Supplement: online supplemental file 1 [file bmjgh-2021-008143supp001.pdf]
